# Rethinking PFAS Behavior in Phosphogypsum Stacks: A Hydrochemically Controlled Multiphase Perspective

**DOI:** 10.3390/molecules31111838

**Published:** 2026-05-27

**Authors:** Zhipeng Du, Kaiyu Shi, Xianghua Yan, Hongbo Zhou, Xingrun Wang

**Affiliations:** 1 State Key Laboratory of Environmental Criteria and Risk Assessment, Chinese Research Academy of Environmental Sciences, Beijing 100012, China; duzp@craes.org.cn (Z.D.); shiky@craes.org.cn (K.S.); yanxh@craes.org.cn (X.Y.); 2 Hydrochina Beijing Engineering Corporation Limited, Beijing 100024, China; 3 Key Laboratory for Water and Sediment Sciences of Ministry of Education, School of Environment, Beijing Normal University, Beijing 100875, China

**Keywords:** phosphogypsum stacks, PFAS, hydrochemical control, multiphase repartitioning, groundwater risk

## Abstract

Phosphogypsum (PG) stacks are traditionally assessed as sources of legacy inorganic contaminants, but the behavior of emerging contaminants in these chemically complex systems remains poorly understood. This opinion article proposes that PFAS, if present in PG stacks, may not be adequately described by partitioning concepts derived from dilute groundwater or ordinary soil porewater systems. Instead, the low-pH, high-ionic-strength, and calcium–sulfate-rich conditions of PG leachate may promote hydrochemistry-mediated repartitioning of PFAS. Under such conditions, PFAS may exhibit reduced apparent aqueous stability, enhanced association with PG particles or colloids, retention on particle surfaces, and enrichment at air–water interfaces, forming potential hidden reservoirs with the potential for delayed release and episodic remobilization. Consequently, dissolved concentrations alone may underestimate total PFAS storage and long-term groundwater risk in and around PG stack systems. Overall, this study highlights the need to shift from conventional dilute-system assumptions toward a hydrochemically mediated multiphase framework for PFAS occurrence assessment, monitoring design, and risk evaluation in phosphogypsum environments and other chemically complex industrial waste systems.

## 1. Background

The rapid expansion of the phosphorus chemical industry has been accompanied by the large-scale generation and open-air accumulation of phosphogypsum (PG), making PG stacks one of the most representative industrial solid-waste storage systems in phosphate-producing regions [1,2]. Environmental investigations and engineering management of PG stacks have long focused on conventional inorganic contaminants in leachate, particularly fluoride, phosphate, sulfate, and associated metals/metalloids, and radionuclide-related constituents released under acidic and highly mineralized conditions [3,4,5]. Accordingly, existing risk assessment frameworks and control strategies for PG stacks have been developed largely on the basis of the source of release, migration, and attenuation behavior of these legacy pollutants [6,7]. In this context, PG leachate is commonly regarded as an acidic, mineralized wastewater, whereas the stack itself is often treated primarily as a long-term source of inorganic contaminant release.

However, this conventional perspective may be incomplete for chemically complex PG systems, especially as concern grows over contaminants of emerging concern in industrial solid-waste environments [8,9]. Per- and polyfluoroalkyl substances (PFAS), a large class of highly persistent anthropogenic chemicals, have attracted increasing attention because of their environmental mobility, resistance to degradation, interfacial activity, and potential for long-term human exposure [10,11,12,13]. In contrast to many traditional inorganic pollutants, PFAS exhibit amphiphilic molecular structures and can be regulated not only by dissolved-phase transport, but also by sorption to solid surfaces, association with colloids, and enrichment at air–water interfaces [14,15,16]. These characteristics raise an important question as to whether PFAS, if introduced into PG stack systems, would behave in the same way as they do in ordinary soil–groundwater environments.

This question is particularly relevant because phosphogypsum leachate does not resemble typical dilute groundwater or soil porewater. Instead, it commonly exhibits low pH, high ionic strength, and elevated concentrations of major ions such as sulfate, calcium, fluoride, and phosphate [5,17]. Such a hydrochemical background may alter PFAS speciation, apparent aqueous stability, solid-associated retention, and air–water interfacial adsorption [18,19,20,21,22,23]. Therefore, the environmental behavior of PFAS in PG stacks cannot be adequately assessed by directly extrapolating from conventional partitioning models developed for dilute aqueous systems [24]. Rather, PG stacks should be reconsidered as hydrochemically active environments in which extreme water chemistry may reshape the occurrence, migration, and release of PFAS [25].

Although systematic field datasets on PFAS in PG stacks remain scarce, preliminary unpublished observations from a large PG stack in southwestern China suggest that PFAS may be detectable in both stack materials and hydraulically connected downgradient groundwater. Detectable PFAS were identified in stack materials at approximately 5 μg/kg and in hydraulically connected downgradient groundwater at approximately 0.5 μg/L. These observations are not sufficient to establish a complete source–pathway–receptor relationship, nor do they provide a comprehensive inventory of PFAS species or mass storage within the stack. Nevertheless, they indicate that PFAS occurrence in PG stack systems deserves further attention. More importantly, they highlight the need to move beyond a purely legacy-inorganic perspective and to examine how PFAS, once present in PG environments, may enter leachate, repartition across multiple phases, and contribute to long-term environmental risk in and around PG storage facilities.

Against this background, this article aims to reframe PG stacks as hydrochemically controlled multiphase systems for PFAS rather than passive repositories of inorganic contaminants alone. Specifically, we discuss the physicochemical properties and environmental significance of PFAS in PG settings, examine potential pathways by which PFAS may enter PG leachate, and propose a hydrochemistry-driven conceptual framework to explain their repartitioning among aqueous, solid-associated, colloid-associated, and air–water interfacial domains. Because direct evidence for PFAS occurrence and transport in PG stacks is still limited, this article should be viewed as a hypothesis-driven perspective rather than a site-specific occurrence study. By doing so, we seek to provide a conceptual basis for future occurrence assessment, monitoring design, and risk evaluation of PFAS in phosphogypsum stack environments.

## 2. PFAS Properties, Health Concerns, and Environmental Risks in Phosphogypsum Stacks

**Figure 1:**
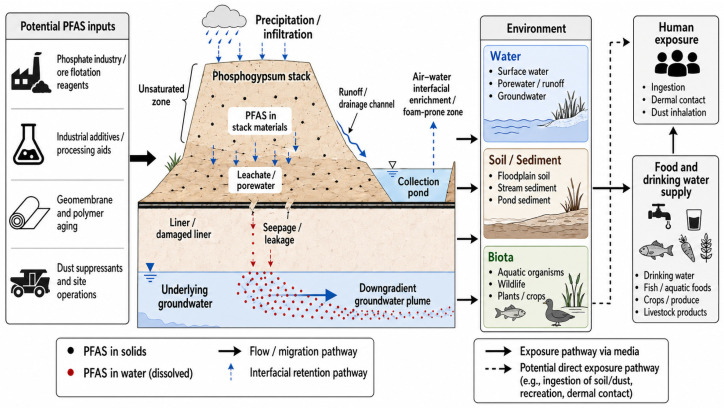
Risk-relevant occurrence and migration of PFAS in phosphogypsum stack systems.

From a health and environmental perspective, PFAS are of concern primarily because of their persistence, mobility, interfacial activity, and potential to cause long-term exposure through water resources [10,11,26]. In PG stack systems, the most relevant issue is therefore not short-term acute toxicity within the stack itself, but the possibility that PFAS, if present, may be retained in stack materials, redistributed by leachate, and gradually released to surrounding groundwater or other connected receptors [8,9,27]. This makes even low-level PFAS occurrence environmentally meaningful when the source is persistent and hydrologically connected to adjacent systems (Figure 1) [14,28].

A further reason for concern is that PFAS may enter the PG stack–leachate system through several potential pathways rather than a single discrete source. One possible pathway is the carryover of PFAS-containing or fluorinated reagents, surfactants, processing aids, or associated organic additives used during phosphate ore beneficiation and industrial production, which may become incorporated into phosphogypsum residues during generation and disposal. Additional potential inputs may arise during stack construction and operation, including the aging and leaching of polymeric materials, geomembranes, coatings, or fluorinated additives used for surface management or dust suppression [14,27]. Once present in the solid matrix, these compounds do not remain environmentally inert, but may become available for subsequent release under infiltration, wetting, and leaching conditions. At present, these pathways should be regarded as plausible input routes rather than fully quantified sources, because systematic source-apportionment studies for PFAS in PG stack environments remain limited.

Rainfall infiltration provides the most direct mechanism by which PFAS associated with PG solids may be transferred into porewater and leachate. As water percolates through the variably saturated stack, PFAS may desorb from solid residues, redistribute between solid and aqueous phases, associate with colloids, or become enriched at air–water interfaces. In addition to vertical infiltration, lateral runoff, internal drainage channels, seepage collection systems, and leachate ponds may further concentrate or redistribute PFAS mass within the stack system (Figure 1). Where liners are damaged, aging, or hydraulically incomplete, part of this PFAS-bearing leachate may migrate downward as seepage and enter the underlying aquifer, thereby creating a potential pathway from the waste body to downgradient groundwater receptors [24,27].

The environmental risk of PFAS in PG stacks may thus differ from that of conventional inorganic pollutants typically considered in PG management [1,2]. For fluoride, phosphate, sulfate, metals/metalloids, and radionuclide-related constituents, risk assessment is usually based on source strength, aqueous concentration, migration distance, and receptor exposure [3,4,5]. For PFAS, however, dissolved concentrations alone may not adequately represent total storage or future release potential because part of the contaminant mass may be temporarily retained within the stack through solid association, colloid interaction, or interfacial enrichment [15,16,18,21]. In this sense, PFAS in PG stacks should be viewed not only as possible co-occurring contaminants, but also as pollutants with the potential for hidden storage, delayed release, and episodic remobilization [14,27].

From a toxicological and public health perspective, PFAS are particularly important because prolonged exposure to trace levels has been associated with adverse outcomes such as immunotoxicity, endocrine disruption, developmental effects, and lipid metabolism disorders [26,29]. For this reason, drinking water advisories and regulatory limits for PFAS are commonly established at the nanogram-per-liter (ng/L) level [13,30]. Within the context of PG stacks, this stringent toxicity threshold changes the risk perspective [30]. A PG stack does not need to release massive quantities of PFAS to cause a severe environmental impact; even slow or intermittent seepage may be important if it is persistent, hydraulically connected to groundwater, and insufficiently captured by monitoring systems [24,27]. Table 1 summarizes the PFAS properties most relevant to PG stack environments. These properties do not imply that all PFAS will behave in the same way. Rather, PFAS behavior is expected to be compound-specific and influenced by chain length, functional headgroup, precursor transformation potential, solution chemistry, and the physical structure of the stack.

Taken together, the environmental significance of PFAS in PG stacks lies not only in their possible detectability, but also in the way they may be introduced into the stack–leachate system, redistributed through infiltration and seepage, retained in nonaqueous domains, and subsequently sustained as a long-term source to surrounding environmental media. This combination of persistence, multiphase repartitioning, and delayed release distinguishes PFAS from the legacy inorganic pollutants traditionally emphasized in PG studies and provides the basis for understanding their hydrochemistry-driven behavior in PG leachate systems.

## 3. Hydrochemistry-Driven Repartitioning of PFAS in Phosphogypsum Leachate

Pollutants in large phosphogypsum (PG) stacks should not be viewed as isolated species undergoing passive release. Rather, they evolve within a chemically aggressive leachate system characterized by acidity, high salinity, and strong electrolyte effects inherited from phosphate rock impurities and wet-process phosphoric acid production. Within such a setting, the environmental behavior of PFAS may deviate from the partitioning patterns typically inferred from dilute groundwater or conventional soil-water systems. We therefore propose that the potential multiphase repartitioning of PFAS in PG-impacted settings is more plausibly interpreted as a hydrochemistry-controlled response, arising from the interaction between the molecular structure of PFAS and the unusual ionic composition of phosphogypsum leachate.

### 3.1. Phosphogypsum Leachate as a Strong Electrolyte Medium

To evaluate the environmental behavior of PFAS in PG stacks, the aqueous phase should first be considered in terms of its distinctive hydrochemical properties. In conventional environmental hydrogeology, shallow groundwater, soil porewater, and many ordinary leachates are commonly treated as relatively dilute solutions in which solute–solute interactions are limited [9,14,31]. In such conventional models, the aqueous phase is often approximated as a transport medium for dissolved contaminants [32]. However, this assumption may be inappropriate for PG stack systems because PG leachate is typically acidic, highly mineralized, and enriched in major ions derived from phosphate rock impurities and wet-process phosphoric acid production [24,33]. Under these conditions, PG leachate can be conceptualized as a chemically active strong-electrolyte medium rather than a passive dilute solution.

The hydrochemical signature of PG leachate is reflected by its low pH and high concentrations of inorganic ions [1,2]. As shown by the unpublished monitoring data from a large PG stack in southwestern China (Table 2), the leachate was acidic, with pH values ranging from 2.25 to 7.00 and an average value of 3.31. It also contained high concentrations of sulfate and calcium, with average concentrations of approximately 1828 mg/L and 2005 mg/L, respectively. Fluoride, phosphate, and ammonium were also detected at measurable to elevated levels [3,17,34]. This specific ionic composition generates an ionic strength that is orders of magnitude higher than that of typical natural freshwaters [35].

From a thermodynamic and physicochemical standpoint, such a high-ionic-strength medium may influence the microscopic solvation environment experienced by amphiphilic organic compounds [25,36]. High concentrations of inorganic ions, particularly multivalent species such as Ca^2+^ and sulfate, can compete for water molecules, modify hydration structures, and reduce the activity of bulk water [18,20,37,38]. These processes may increase the energetic cost for some organic molecules to remain fully solvated in the aqueous phase.

For PFAS, this does not necessarily imply precipitation or complete removal from water, but it suggests that the apparent stability of PFAS in bulk solution may be altered under strongly mineralized conditions. In addition, the acidity and ionic composition of PG leachate may regulate mineral surface charges, compress electrical double layers, and modify fluid–fluid interfacial properties. These effects are particularly relevant for PFAS because their transport and retention can be influenced by electrostatic interactions, cation composition, surface activity, and air–water interfacial adsorption [4,5,39,40]. Therefore, PG leachate should not be treated simply as a carrier of PFAS mass. Rather, it may act as an active hydrochemical medium that modifies PFAS distribution among aqueous, solid-associated, colloid-associated, and air–water interfacial domains.

### 3.2. Reduced Apparent Aqueous Stability of PFAS Under High Ionic Strength

Once PG leachate is recognized as a high-ionic-strength electrolyte medium, the next question is how such a background may alter the behavior of PFAS in the aqueous phase. In dilute groundwater or ordinary soil porewater, PFAS are often conceptualized primarily as dissolved contaminants whose transport can be interpreted mainly through aqueous advection, dispersion and sorption, and interfacial retention [14,41]. However, this assumption may become less appropriate in phosphogypsum leachate, where the elevated ionic burden can modify both water structure and solute activity [36,42]. Under such conditions, some PFAS may exhibit reduced apparent aqueous stability and an increased tendency to repartition toward interfaces, particles, or colloidal domains [16,43].

**Figure 2:**
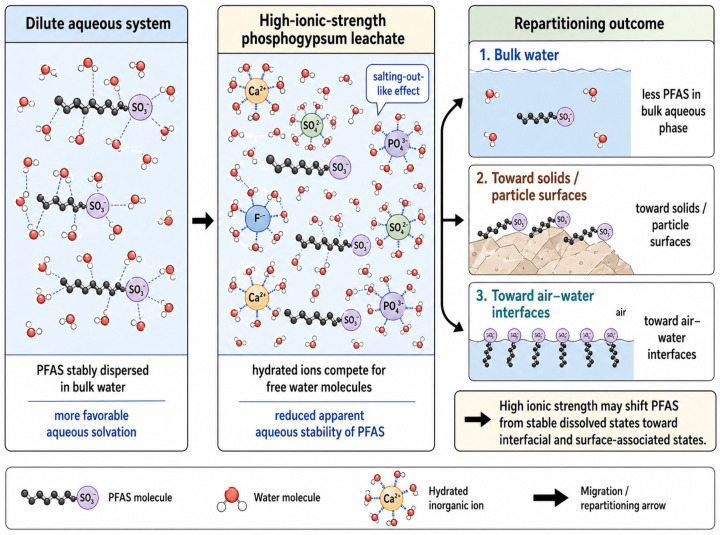
Conceptual illustration of reduced aqueous stability and salting-out-like repartitioning of PFAS under high-ionic-strength phosphogypsum leachate conditions. Compared with dilute aqueous systems, the strong electrolyte background may reduce PFAS stability in bulk water and promote redistribution toward particle surfaces and air–water interfaces.

A possible explanation for this shift is a salting-out-like effect induced by the strong electrolyte background (Figure 2) [25]. In highly mineralized solutions, strongly hydrated inorganic ions compete for water molecules and alter the solvation environment of amphiphilic organic compounds. For PFAS, this should not be interpreted as classical precipitation from solution. Rather, it suggests that the thermodynamic favorability of remaining in the bulk aqueous phase may decrease as ionic strength increases [24,44]. As a result, PFAS may become more likely to redistribute from the aqueous phase and redistribute to air–water interfaces, particle surfaces, or other energetically favorable domains [21,44].

This effect may be particularly relevant in phosphogypsum leachate because the aqueous matrix is not only saline, but also acidic and enriched in multivalent ions. Compared with monovalent electrolytes alone, divalent cations such as Ca^2+^ can exert stronger influences on the local electrostatic environment and may reduce electrostatic repulsion between anionic PFAS and mineral surfaces [18,45]. At the same time, the coexistence of sulfate, fluoride, and phosphate contributes to a chemically crowded solution environment in which PFAS behavior is governed not simply by their intrinsic molecular properties, but by the combined effect of ionic strength, ion composition, and surface chemistry [17,23]. As a result, PFAS in phosphogypsum leachate should not be interpreted solely through conceptual models developed for dilute aqueous systems.

The reduction in apparent aqueous stability is unlikely to be uniform across all PFAS species. The response may vary with chain length, functional headgroup, precursor chemistry, and molecular structure [24,46]. Longer-chain compounds or those with stronger surface activity may be more prone to repartition from bulk water under high-salinity conditions, whereas shorter-chain compounds may remain relatively more mobile in water [41,46]. This implies that high ionic strength does not simply reduce PFAS concentrations in solution, but may instead reshape the relative distribution of different PFAS among dissolved, surface-associated, colloid-associated, and interfacial states.

From a conceptual standpoint, the key implication is that high ionic strength may shift the role of the aqueous phase from a dominant storage compartment to a transitional medium within a broader multiphase partitioning system [43,44]. Under such conditions, measured dissolved concentrations may represent only part of the total PFAS inventory present in the phosphogypsum stack environment [14,41]. A decrease in apparent aqueous stability may therefore provide a mechanistic link between the hydrochemical background of PG leachate and the solid-associated retention and air–water interfacial enrichment discussed below.

### 3.3. Solid-Associated Retention and Air–Water Interfacial Enrichment

**Figure 3:**
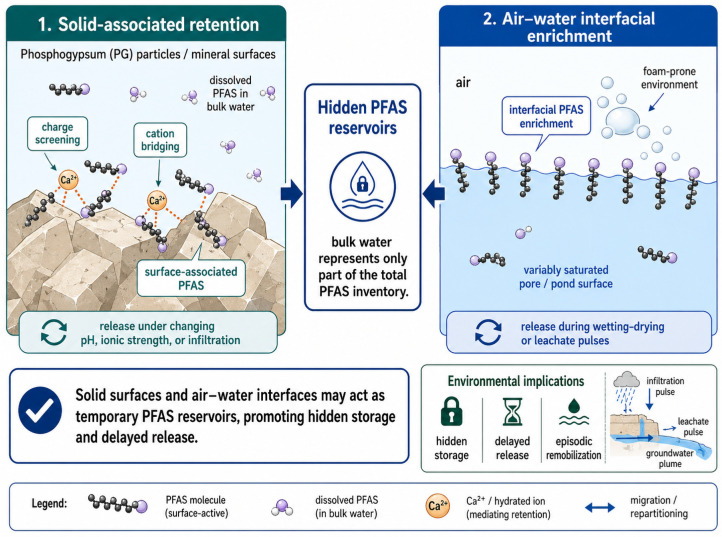
Conceptual illustration of solid-associated retention and air–water interfacial enrichment of PFAS in PG stack systems. In PG leachate, PFAS may be retained on PG particle and mineral surfaces through charge screening and Ca^2+^-mediated cation bridging, and may also accumulate at air–water interfaces in variably saturated pores or pond surfaces. These solid and interfacial domains may act as potential hidden PFAS reservoirs, promoting delayed release and episodic remobilization during changes in pH, ionic strength, infiltration, or leachate pulses.

A direct consequence of reduced apparent aqueous stability under high-ionic-strength conditions is that PFAS may increasingly repartition from bulk water to other environmentally relevant domains [14,46]. In phosphogypsum (PG) leachate systems, two domains are particularly important: solid surfaces associated with PG particles and secondary mineral phases, and air–water interfaces present in variably saturated pores, drainage pathways, and surface leachate features. Together, these domains may act as temporary storage compartments that regulate PFAS retention, remobilization, and long-term release (Figure 3) [47].

Solid-associated retention may be enhanced in PG environments because the stack matrix provides abundant mineral and particle surfaces for sorption [15,48]. In addition to direct surface interactions, the hydrochemical background of PG leachate may further modify this retention. Elevated ionic strength can compress the electrical double layer and reduce electrostatic repulsion between anionic PFAS headgroups and mineral surfaces. High concentrations of divalent cations, particularly Ca^2+^, may also promote charge-screening or cation-bridging effects that facilitate PFAS association with particle surfaces [48,49]. Under such conditions, part of the PFAS mass may be retained in solid-associated forms rather than remaining freely dissolved in the aqueous phase.

This process is especially important in PG stacks because the solid matrix is the dominant structural component of the system. PFAS associated with particle surfaces may therefore constitute an internal reservoir that is not captured adequately by bulk leachate measurements alone. Such retention is unlikely to be static. Changes in ionic composition, water saturation, pH, or infiltration intensity may destabilize previously retained PFAS and remobilize them into porewater or seepage pathways [47,50]. Solid-associated retention in PG stacks should therefore be viewed as a dynamic process contributing both to temporary storage and delayed release.

Air–water interfacial enrichment provides another important mechanism for PFAS retention in PG stack systems [46,48]. Because PFAS are amphiphilic and surface-active, they tend to accumulate at fluid interfaces rather than remaining uniformly distributed in bulk water. This process may be particularly relevant in PG environments, where variably saturated pore spaces, entrapped air, transient seepage zones, drainage channels, collection ponds, and local foam-prone areas can create substantial air–water interfacial area [47]. Under these conditions, the air–water interface may function as an additional storage domain for PFAS within and around the stack system [48].

The importance of this interfacial domain may be further amplified by the hydrochemical conditions of PG leachate. Previous studies have shown that PFAS adsorption at air–water interfaces can increase with ionic strength and may be sensitive to the presence of divalent cations [48,49]. In a saline and calcium-rich leachate environment, PFAS may therefore display stronger interfacial enrichment than would be expected in dilute groundwater systems. This suggests that part of the PFAS inventory in PG stacks may reside not in the dissolved phase, but at interfaces that are spatially distributed throughout the unsaturated zone and surface leachate features [46,50].

Taken together, solid-associated retention and air–water interfacial enrichment indicate that PFAS in PG stack systems should be understood within a multiphase retention framework rather than purely as dissolved contaminants. These two domains may jointly reduce the fraction of PFAS measured in bulk water while increasing the potential for hidden storage and delayed release. However, their relative importance is expected to vary with PFAS species, leachate chemistry, stack saturation, and weathering conditions.

### 3.4. Multiphase Repartitioning and Implications for Release

The hydrochemical characteristics of PG leachate suggest that PFAS release from stack systems may be controlled by a dynamic balance among dissolved, solid-associated, colloid-associated, and air–water interfacial states. Under acidic and high-ionic-strength conditions, PFAS may not remain in a single stable form, but may shift among these domains as leachate chemistry, water saturation, and flow conditions change.

This multiphase behavior has direct implications for contaminant release and monitoring. If part of the PFAS inventory is temporarily retained on PG particles, associated with colloids, or enriched at air–water interfaces, dissolved concentrations in leachate may represent only a fraction of the total mass stored within the stack. Previously retained PFAS may later be remobilized by changes in pH, ionic strength, water saturation, infiltration intensity, or leachate composition. As a result, PFAS release from PG stacks may occur not only through continuous dissolved phase transport, but also through delayed or episodic mobilization from retained reservoirs.

Importantly, this framework does not imply that all PG stacks will exhibit the same PFAS partitioning behavior. Older, weathered, or neutral-pH zones within a stack may show weaker electrolyte effects and different PFAS retention–release behavior than fresh, acidic, high-ionic-strength leachate. Therefore, site age, hydrochemical gradients, stack construction, saturation conditions, and operational history should be considered when applying this framework to field investigations.

## 4. Implications for Risk Assessment and Future Research

The central implication of the above framework is that the fate and risk of PFAS in PG stacks may be shaped not only by PFAS molecular structure or source strength, but also by the hydrochemical conditions created by legacy inorganic constituents. Sulfate, calcium, fluoride, phosphate, acidity, and elevated ionic strength should therefore not be viewed merely as co-occurring background conditions. Instead, they may influence whether PFAS remain mobile in the aqueous phase, become associated with PG particles or colloids, or accumulate at air–water interfaces. This shifts the conceptual model of PG contamination from simple co-occurrence of inorganic and organic contaminants towards hydrochemically mediated coupling between legacy pollutants and contaminants of emerging concern.

This perspective has important implications for risk assessment. Existing PG evaluation frameworks have largely focused on fluoride, phosphate, sulfate, metals/metalloids, and radionuclide-related risks, emphasizing source of release intensity, dissolved concentrations, plume migration, and receptor exposure. However, for PFAS and other interracially active contaminants, dissolved-phase concentrations alone may not adequately capture environmental risk. In highly mineralized PG leachate, PFAS occur as a dynamic mixture of dissolved, particle-associated, colloid-associated, and air–water interfacial fractions. Future identification and evaluation of emerging contaminants in PG stacks should therefore address not only whether PFAS are detected, but also in what forms they occur, where they are retained, and under what hydrochemical or hydrological conditions they may be released.

A simple mass-balance perspective illustrates why this distinction is important. A low dissolved PFAS concentration in leachate does not necessarily indicate a low total PFAS inventory if a larger fraction of the mass is stored on PG solids, associated with colloids, or enriched at air–water interfaces. Conversely, episodic changes in pH, ionic strength, saturation, or infiltration intensity may remobilize previously retained PFAS and generate delayed concentration pulses in seepage or groundwater. Therefore, monitoring programs based only on dissolved PFAS concentrations in leachate or groundwater may underestimate the long-term release potential of PG stacks (Table 3).

At the same time, this proposed framework remains a hypothesis-driven research direction rather than a fully established mechanism. Systematic evidence for PFAS occurrence, source apportionment, and input fluxes in PG stacks is still limited. At present, PFAS in PG systems are more defensibly described as potentially co-occurring contaminants than as process-specific signature pollutants. Second, the relative importance of salting-out-like effects, cation bridging, enhanced interfacial adsorption, and colloid-facilitated retention likely varies with PFAS chain length, functional headgroup, precursor chemistry, site-specific leachate composition, stack age, weathering status, and hydrological regime. Therefore, the proposed framework should be tested through targeted laboratory experiments, column studies, and field-scale mass-balance monitoring.

The broader relevance of this perspective extends beyond a single country or management context. Although PG investigation and remediation are particularly urgent in regions with intensive phosphate fertilizer production, the underlying issue is international: many PG stacks worldwide are large, long-lived, chemically reactive, and hydrologically connected waste systems. Similar concerns may also apply to other acidic, saline, or chemically complex industrial solid-waste environments where conventional dilute-solution assumptions may not adequately describe the behavior of emerging contaminants. Reframing PG stacks as hydrochemically controlled multiphase systems may therefore provide a useful conceptual basis for PFAS assessment, monitoring design, and risk management in global phosphate-production regions and related industrial waste settings.

## 5. Conclusions

Phosphogypsum (PG) stacks should be reconsidered not only as long-term sources of legacy inorganic pollutants, but also as hydrochemically active multiphase systems that may regulate the environmental behavior of PFAS. Under the low-pH, high-ionic-strength, and calcium–sulfate-rich conditions of PG leachate, PFAS may not be represented adequately by the dissolved phase alone, but may repartition among aqueous, solid-associated, colloid-associated, and air–water interfacial domains. This behavior implies that dissolved concentrations alone may underestimate total PFAS storage, delayed release potential, and long-term groundwater risk in and around PG stack systems. Overall, a shift from conventional dilute-system assumptions toward a hydrochemically mediated multiphase framework is essential for future PFAS occurrence assessment, monitoring design, and risk evaluation in phosphogypsum stack environments.

The key contribution of this opinion article is to propose a hydrochemistry-mediated multiphase framework for understanding PFAS behavior in PG environments. This framework shifts attention from simple dissolved-phase transport to the coupled effects of leachate chemistry, solid-associated retention, colloid interaction, and air–water interfacial enrichment. Because systematic field and experimental evidence remain limited, future studies should test this framework through targeted laboratory experiments, column leaching tests, and field-scale mass-balance monitoring.

## Figures and Tables

**Table 1:** PFAS properties most relevant to environmental risk in phosphogypsum stack systems.

Property	General Environmental Significance	Relevance in Phosphogypsum Stack Systems
Persistence	PFAS are highly resistant to chemical, thermal, and biological degradation and can remain in environmental media for long periods [10,11].	Once introduced into phosphogypsum stacks, PFAS may persist in stack materials, porewater, and leachate rather than being readily attenuated [8,9].
Aqueous mobility	Many PFAS can migrate with infiltrating water and groundwater flow, especially under low retardation conditions [14,28].	PFAS may be transported by rainfall-induced leachate, seepage, and downgradient groundwater flow from phosphogypsum stacks [17,27].
Surface activity/interfacial affinity	PFAS tend to accumulate at air–water interfaces and, in some cases, at solid surfaces rather than remaining only in the dissolved phase [15,16].	In variably saturated stack systems, PFAS may partition to air–water interfaces, foam-prone zones, leachate ponds, drainage channels, and particle surfaces [21,22].
Structural heterogeneity	PFAS behavior varies with carbon-chain length and functional headgroup, leading to compound-specific transport and retention [23,24].	Different PFAS may show different migration, retention, and repartitioning behavior in phosphogypsum leachate and stack materials [18,23].
Sensitivity to hydrochemical conditions	PFAS partitioning can be influenced by ionic strength, pH, cation composition, and organic/mineral surfaces [20,24].	The acidic, calcium–sulfate-rich, and high-ionic-strength leachate of PG stacks may enhance PFAS repartitioning and alter their apparent aqueous stability [21,25].

**Table 2:** Conventional hydrochemical composition of leachate from a large phosphogypsum stack.

No.	Indicator	Sample Count	Minimum Value	Maximum Value	Average Value	Median Value
1	pH	35	2.25	7	3.31	2.7
2	NH^4+^	35	1.16	11.2	4.079	3.56
3	F^−^	35	5.7	286	79.89	72.4
4	TP	35	5.46	987.5	429.3	430
5	SO_4_^2−^	35	986	4320	1828	1760
6	Ca^2+^	35	1050	4560	2005	1820

Note: Except for pH, all concentrations are expressed in mg/L. The values represent the range, average, and median of 35 leachate samples collected from a large PG stack in southwestern China. These unpublished monitoring data are used only to describe the hydrochemical background of PG leachate. PFAS concentrations in leachate were not included in this table.

**Table 3:** Illustrative mass-balance implication of multiphase PFAS storage in PG stacks.

Compartment	Example Concentration	Example Amount of Medium	Estimated PFAS Mass	Implication
Dissolved leachate	0.5 μg/L	1000 m^3^ leachate	0.5 g	Represents only the water-phase inventory
Solid-associated PG material	5 μg/kg	10,000 t PG material	50 g	A low solid-phase concentration may correspond to a much larger stored mass
Colloid-associated/interfacial domains	Not routinely measured	Site-specific	Unknown	May contribute to hidden storage and delayed release

Note: The values in this table are illustrative and are used only to demonstrate the mass-balance implication of multiphase PFAS storage. They should not be interpreted as a site-specific PFAS inventory.

## Data Availability

No new data were created or analyzed in this study. Data sharing is not applicable.
